# Aqueous Corrosion of Aluminum-Transition Metal Alloys Composed of Structurally Complex Phases: A Review

**DOI:** 10.3390/ma14185418

**Published:** 2021-09-19

**Authors:** Libor Ďuriška, Ivona Černičková, Pavol Priputen, Marián Palcut

**Affiliations:** Faculty of Materials Science and Technology in Trnava, Institute of Materials Science, Slovak University of Technology in Bratislava, 917 24 Trnava, Slovakia; libor.duriska@stuba.sk (L.Ď.); ivona.cernickova@stuba.sk (I.Č.); pavol.priputen@stuba.sk (P.P.)

**Keywords:** aluminum, transition metal, corrosion, quasicrystal, approximant

## Abstract

Complex metallic alloys (CMAs) are materials composed of structurally complex intermetallic phases (SCIPs). The SCIPs consist of large unit cells containing hundreds or even thousands of atoms. Well-defined atomic clusters are found in their structure, typically of icosahedral point group symmetry. In SCIPs, a long-range order is observed. Aluminum-based CMAs contain approximately 70 at.% Al. In this paper, the corrosion behavior of bulk Al-based CMAs is reviewed. The Al–TM alloys (TM = transition metal) have been sorted according to their chemical composition. The alloys tend to passivate because of high Al concentration. The Al–Cr alloys, for example, can form protective passive layers of considerable thickness in different electrolytes. In halide-containing solutions, however, the alloys are prone to pitting corrosion. The electrochemical activity of aluminum-transition metal SCIPs is primarily determined by electrode potential of the alloying element(s). Galvanic microcells form between different SCIPs which may further accelerate the localized corrosion attack. The electrochemical nobility of individual SCIPs increases with increasing concentration of noble elements. The SCIPs with electrochemically active elements tend to dissolve in contact with nobler particles. The SCIPs with noble metals are prone to selective de-alloying (de–aluminification) and their electrochemical activity may change over time as a result of de-alloying. The metal composition of the SCIPs has a primary influence on their corrosion properties. The structural complexity is secondary and becomes important when phases with similar chemical composition, but different crystal structure, come into close physical contact.

## 1. Introduction

Aluminum alloys are frequently used materials in many applications due to their low specific mass, good mechanical properties, formability, high recycling potential and superior corrosion resistance [1,2]. Two principal classifications of Al alloys exist: wrought and casting alloys, both of which are heat-treatable and non-heat-treatable. Alloying elements, including transition metals (TM), are often added to Al alloys to improve their physical and mechanical properties [3]. Complex metallic alloys (CMAs) are materials composed of structurally complex intermetallic phases (SCIPs). In SCIPs a long-range order is observed. Well-defined atomic clusters are found in the structure, typically of icosahedral symmetry. Such phases also include quasicrystals (QCs) characterized by an infinitely large unit cell (often with icosahedral symmetry) and quasicrystalline approximants, whose unit cell can be described by “classical” crystallography but contains hundreds to thousands of atoms [4,5,6,7,8]. Aluminum-based CMAs contain approximately 70 at.% Al. Most Al-based CMAs are alloyed with TM. However, non-transition metals like Mg, can also be used. Depending on their chemical composition, the structural complexity may vary from dozens of atoms per unit cell up to thousands of atoms. This group of materials has been receiving a significant attention since the discovery of quasicrystals in melt-spun Al–Mn alloy by Shechtman et al. [4]. The discovery of quasicrystals was awarded by Nobel prize in Chemistry in 2011.

**Figure 1:**
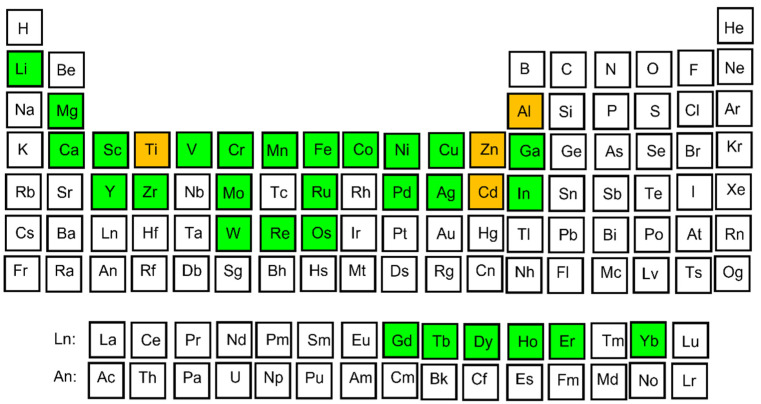
Chemical elements forming thermodynamically stable QCs. Main forming elements (Al, Ti, Zn, Cd) are marked by orange color, alloying elements are denoted by green.

In 1982, D. Shechtman discovered a quasiperiodic arrangement of atoms in an Al–Mn alloy showing an icosahedral symmetry but no unit cell [4]. Many such compounds correspond to stable or metastable states in various phase diagrams [5]. The icosahedral phase, as the first quasicrystalline structure discovered in the Al–Mn system, has a long-distance arrangement without translational symmetry [4,5,6,7]. Many elements in thermodynamically stable QCs observed yet belong to alkali, alkali-earth, transition, or rare-earth metals (Figure 1). Typical examples of QCs and quasicrystalline approximants are Al–TM alloys, where the TM is formed by one or more transition metals (TM = Cu, Co, Ni, Fe, etc.) [9]. Thus, the quasicrystalline materials are often low-cost materials that are easy to produce in large amounts [5,6,7,9]. Examples of quasicrystalline phases are summarized in Table 1 [7,8,10,11,12,13,14,15,16,17,18,19,20,21,22,23,24].

The atomic structure of QCs offers an interesting combination of properties, such as low thermal conductivity combined with high electrical resistivity [25], low coefficient of friction [26,27] and/or high hardness [28,29,30]. The CMAs have a large potential for technical applications as the combination of the properties is unique and not observed in conventional materials. Potential applications in thermoelectrics, coatings, composites, and catalysts have been reported [5,9,31]. It has been shown that quasicrystalline materials may be more efficient catalysts compared to their crystalline counterparts. In 1994, a superior catalytic activity of quasicrystalline Al–Pd alloy relative to crystalline Al–Pd, pure Pd, and pure Cu was described [32]. Surface energies of icosahedral Al_70_Pd_21_Mn_9_ and Al_65_Cu_23_Fe_12_ phases are comparable to polytetrafluoroethylene, and yet quite different from pure aluminum and related crystalline materials. Studies revealed that surface energy is decreasing by increasing structural perfection [33]. Considering the above fact, a direct application of the quasicrystalline coatings as scratch resistant films is already on the market, offering a lowered adhesion to some polymers or food [5].

Other possible applications of QCs are found in energy saving, namely thermal insulation, light absorption, power generation, and hydrogen storage [34]. Thermal barrier demonstrators could be assessed in real conditions during the aircraft engine test on the ground. The design of selective quasicrystalline light absorbers takes advantage of the specific optical properties, e.g., the Al_65_Cu_23_Fe_12_ alloy has solar absorbance of 90% [9,34].

The corrosion of Al-based CMAs is a relatively new field, with first investigations emerging 28 years ago. Initial studies were focused on a small family of alloys. First, corrosion parameters were reported for quasicrystalline Al–Cr–Cu–Fe and Al–Cu–Fe alloys in aqueous Na_2_SO_4_ (0.5 mol dm^−3^) and in solutions of different pH [35]. The corrosion behavior of the quasicrystalline Al–Pd–Mn alloy was later studied in aqueous NaCl [36]. Nevertheless, the experimental polarization curves have not been analyzed in terms of electrochemical reactions. In later years, a more systematic approach to corrosion of Al–based CMAs has been adapted. Alloys with carefully chosen chemical composition and phase constitution have been prepared and investigated [37]. Furthermore, different electrolytes were studied [37,38]. A good thermodynamic stability of the materials at pH between 4 and pH 9 has been observed [37]. Several authors found that the electrochemical properties of the materials are determined by their chemical composition rather than by their complex crystal structure [37,39]. A high temperature oxidation of several Al-based CMAs was also studied [40,41,42]. The presence of high Al concentration improves the corrosion resistance of SCIPs [43,44]. Despite their practical potential, however, the corrosion behavior of Al–TM SCIPs has not been systematically reviewed yet. In the present work, we aim to provide a systematic review of aqueous corrosion behavior of bulk Al-based CMAs and compare them with traditional alloys.

## 2. Crystal Structure of Quasicrystals and Their Approximants

The discovery of QCs changed the view of the composition of solids and prompted a change in the definition of crystals. QCs contain such types of atomic arrangements whose symmetry does not correspond to the “classical” rules of filling the three-dimensional space with building units. The quasicrystalline arrangement contains a five-fold axial symmetry, which was originally considered to be forbidden in classical crystallography. The original definition, which specified the crystal as a material with a regular periodic arrangement of building units, was changed after the discovery of QCs. At present, the crystal is defined as a material with discrete diffraction peaks or as a material with a point diffraction pattern, respectively [4,6,7,8,45].

**Figure 2:**
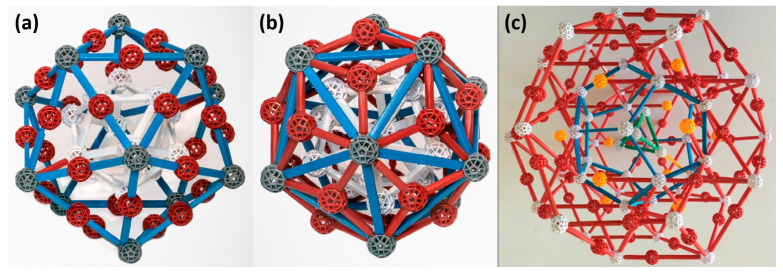
Models of Mackay (**a**), Bergman (**b**) and Tsai (**c**) clusters, reproduced from reference [46].

The building blocks of QCs are mostly arranged in clusters. The clusters are composed of several layers that are formed by individual atoms. Three main types of clusters are known: Mackay, Bergman, and Tsai (Figure 2). The clusters differ from one another in the different layering [4,5,6,7,8,9,46]. Clusters are composed of several shells. In the following lines, three shells will be considered to describe both Mackay and Bergman clusters for the sake of simplicity. The Mackay cluster (Figure 2a) with 54 atoms consists of an inner icosahedron (12 atoms) followed by an icosidodecahedron with 30 atoms. The third shell is an icosahedron with 12 atoms. The Bergman cluster (Figure 2b) differs from the Mackay cluster in the second shell that is formed by a dodecahedron with 20 atoms. Thus, three shells of the Bergman cluster consist of 44 atoms. The Tsai cluster (Figure 2c) has five shells with 158 atoms. The first tetrahedron shell with four atoms sits inside a dodedahedron (20 atoms). The dodecahedron is encased in an icosahedron (12 atoms). The icosahedron sits inside an icosidodecahedron with 30 atoms. The fifth shell is a rhombic triacontahedron (32 atoms + 60 atoms in its edges) [46].

A one-dimensional Fibonacci sequence constitutes the simplest case of quasicrystalline arrangement [47]. In the Fibonacci sequence the sum of the two preceding numbers forms the next number. The ratio of two consecutive numbers is close to τ = (1 + √5)/2 = 1.61803398875 and is called the “golden ratio”. If two segments are selected, with L representing a longer segment and S a shorter segment, then to form the quasicrystalline arrangement, the shorter segment S is replaced by a longer segment L and the combination SL replaces the longer segment L. This results in a following sequence

S → L → SL → LSL → SLLSL → LSLSLLSL → SLLSLLSLSLLSL(1)

**Figure 3:**
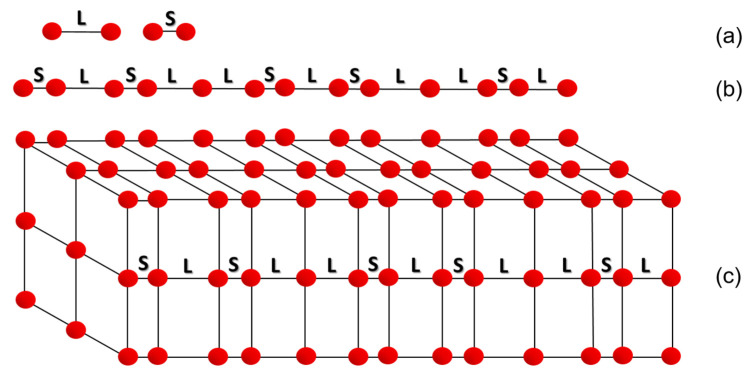
One-dimensional quasicrystalline arrangement: structural segments (**a**), one-dimensional quasiperiodicity (**b**), and simple quasicrystal in three-dimensional space (**c**).

The sequence (1) has features of order, but it is not periodically ordered. There are sections SL and SLL, which alternate but do not repeat with regular periodicity. In fact, there is no periodically repeating segment, a so-called unit cell, in the Fibonacci sequence [6,47]. Based on the Fibonacci sequence, it is possible to draw a one-dimensional quasicrystal graphically (Figure 3). In Figure 3a, letters S and L correspond to shorter and longer segments used in the Fibonacci sequence, respectively. Figure 3b shows the arrangement of S and L segments according to the Fibonacci sequence. If the points dividing the line into segments are atoms, a lattice with the quasicrystalline arrangement in one dimension can be obtained. By adding the second and third dimensions, a simple example of a quasicrystalline lattice can be drawn (Figure 3c).

The quasiperiodic arrangement in two-dimensional space can be represented by Penrose tiling [9,45]. This arrangement consists of two tiles that do not repeat periodically in two-dimensional space. The construction is given by two basic units: a wider rhombus with acute angle α = 2π/5 and a narrower rhombus with acute angle α = π/5. These tiles are arranged in shapes with a five-fold axis of symmetry. As with the Fibonacci sequence, the Penrose tiling is not arranged completely randomly. Basic repeating motifs, such as a star with a 5-axis axis of symmetry, are found in the arrangement of tiles, but are not repeated periodically. Such a star in the Penrose tiling may correspond to a cluster in a real quasicrystalline arrangement.

In three-dimensional space, two types of rhombohedra form the three-dimensional quasicrystalline arrangement containing elements with the icosahedral symmetry. This arrangement is called icosahedral. In the structure of QCs, no unit cell repeating regularly can be found. Thus, its size is theoretically infinite. In fact, the size of the whole grown crystal is the size of the cell. These large unit cells are in contrast to other metallic materials, whose unit cells are built from small number of atoms [48].

Two-dimensional QCs containing a quasicrystalline arrangement along two axes (in one plane) can be further divided based on crystallographic rules of symmetry, which are based on their diffraction patterns. There are octagonal QCs (O-type) with eight-fold rotational symmetry, decagonal (D-type) with ten-fold rotational symmetry, and dodecagonal (DD-type) with twelve-fold rotational symmetry [12]. The three-dimensional QC, also called icosahedral QC (i-QC), is quasiperiodic along all three axes [49]. The clusters usually comprise one of the icosahedral–shaped layers, or the entire clusters can be arranged in the icosahedral shape. The presence of five-fold axes of symmetry in the icosahedral structure is related to the point diffraction spectrum of the i-QC showing ten-fold symmetry.

**Figure 4:**
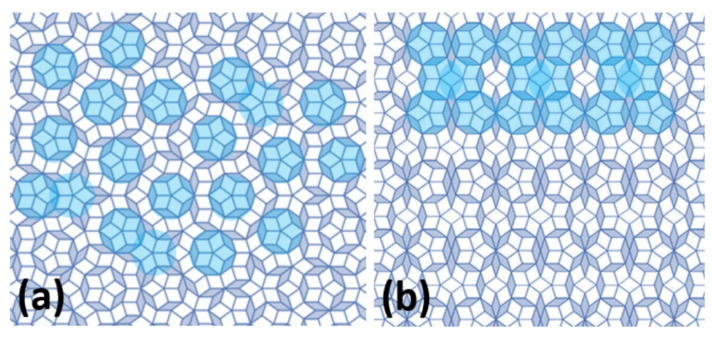
Schematic structure of quasicrystal (**a**) and quasicrystalline approximant (**b**) with denoted star-shape corresponding to clusters in real structure. Quasicrystalline arrangement is represented by Penrose tiling.

In addition to QCs, there are also arrangements with many atoms in the lattice along with the presence of a cluster-based structure. The arrangements are called quasicrystalline approximants. The QCs and the quasicrystalline approximants (schematic representation given in Figure 4, [50,51]) may consist of equally formed clusters. While QCs have the clusters arranged quasiperiodically in space (i.e., non-periodically), the quasicrystalline approximants have clusters arranged with regular periodicity. Therefore, a unit cell is present in the structure of the quasicrystalline approximant, which is periodically repeated in three-dimensional space. However, a cluster in a quasicrystalline approximant structure may also comprise icosahedron-shaped layers or other shapes with the presence of five- or ten-fold axis of symmetry, and thus their structure may exhibit a diffraction pattern with a hint of a ten-fold axis of symmetry despite the regular arrangement.

**Figure 5:**
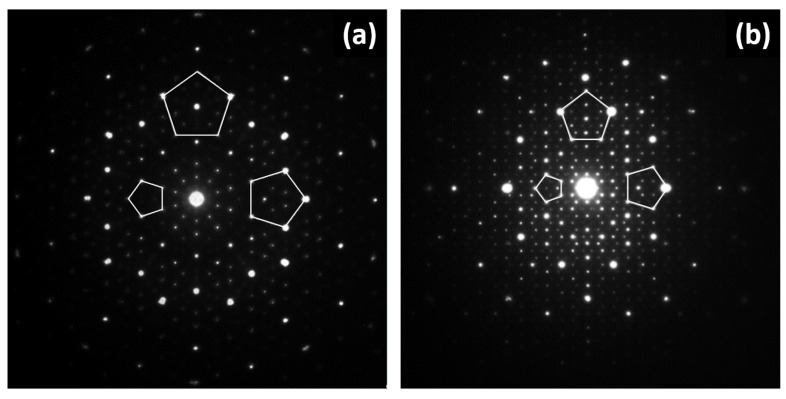
Comparison of electron diffraction patterns in [010] zone axis: decagonal quasicrystal in Al–Co-Cu system (**a**), ε_16_ decagonal quasicrystalline approximant in Al–Pd–Co system (**b**).

In Figure 5, the comparison of electron diffraction patterns of both QC and quasicrystalline approximant is shown. The electron diffraction pattern of the QC (Figure 5a) has a perfect five-fold symmetry. The electron diffraction pattern of a quasicrystalline approximant (Figure 5b) with an orthorhombic unit cell has either two-fold or four–fold axis of symmetry, but there are indications of five-fold symmetry related to the icosahedral arrangement of atoms in clusters [10,11,52,53,54,55,56,57,58,59].

The surface structure of SCIPs is significantly less understood compared to bulk [60,61]. Preliminary results show that the adsorption of small, covalently bonding molecules on icosahedral quasicrystals is very similar to that of pure Al substrate. This is consistent with other studies, which indicate that the surface termination of most SCIPs is Al-rich [60]. A scanning tunneling microscopy (STM) has been utilized to study the surfaces of Al–Pd–Mn quasicrystals [62,63,64]. The STM permits a visualization of the local atomistic surface structure. Specific planes of the bulk structure have been observed as surface terminations [63]. The termination planes are characterized by high atomic density and include elements with the lowest surface energy. Nevertheless, the interpretation of individual STM images is challenging and often needs to be accompanied by theoretical models of the surface [61]. Therefore, ab initio density functional theory (DFT) calculations have been utilized to model quasicrystalline surfaces [65,66]. To perform the calculations, a Vienna ab initio simulation package (VASP) has been used [66]. The atomic structure model of the five-fold Al–Pd–Mn surface is derived from the icosahedral approximant model. In the model, the surface was cut perpendicular to one of its pseudo-five-fold axes. The cleavage position was selected to create high density surface layers consistent with experimental findings. The resulting surface structure is characterized by Penrose tiling [65]. Most tiling vertices coincide with the center of Bergman clusters.

## 3. Overview of Electrochemical Corrosion

The corrosion is a natural process that occurs when metallic materials are exposed to aqueous environments [67,68]. When a metal is immersed in aqueous solution, its cations spontaneously evolve on the metal–electrolyte interface and pass into the solution. During reaction, the material microscopically dissolves. Along with the cations, electrons are also released, and an electrical double layer is created at the metal–electrolyte interface [67]. The release of electrons causes the metal to become electrically charged. As a result of reaction, an electrode potential is established on the metal–electrolyte interface. After some time of immersion, an equilibrium is restored at the electrolyte–metal interface.

**Figure 6:**
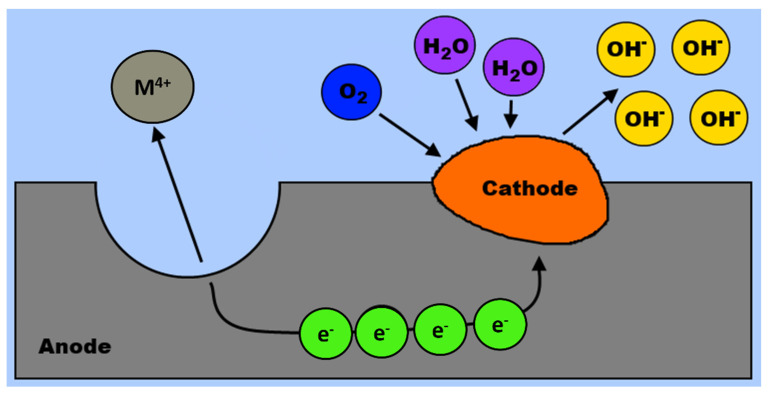
An elementary electrochemical corrosion cell on metal surface.

An overview of the metal corrosion is presented in Figure 6. The corrosion leads to an oxidation of metal and transfer of metal cations to the electrolyte. The oxidation occurs on a metal surface at a specific site known as an anode (anodic reaction site, [67]). Electrons that are released by metal are subsequently consumed by either dissolved oxygen or hydrogen cations in the electrolyte. The reduction reaction takes place at cathode (cathodic reaction site). The relative sizes and locations of cathodic and anodic sites are important variables influencing the overall corrosion rate. The sizes of cathodic and anodic areas may vary greatly; from atomic scales to macroscopically large dimensions.

The metal oxidation is given by the following reaction

(2) $$M\;\to M^{z+}+ze^{-}$$

The Gibbs free energy change ($\Delta G_{r}$) of the reaction is given as

(3) $$\Delta G_{r}=-zFE$$

In this equation, *z* is the number of electrons involved in the reaction, *F* is a Faraday’s constant (96 481 C mol^−1^), and *E* is the electrode potential. At standard conditions (T = 298.15 K, *p* = 101 325 Pa), the standard Gibbs free energy of the reaction ($\Delta G_{r}^{0}$) is related to the standard electrode potential (*E*^0^)

(4) $$\Delta G_{r}^{0}=-zFE^{0}$$

**Figure 7:**
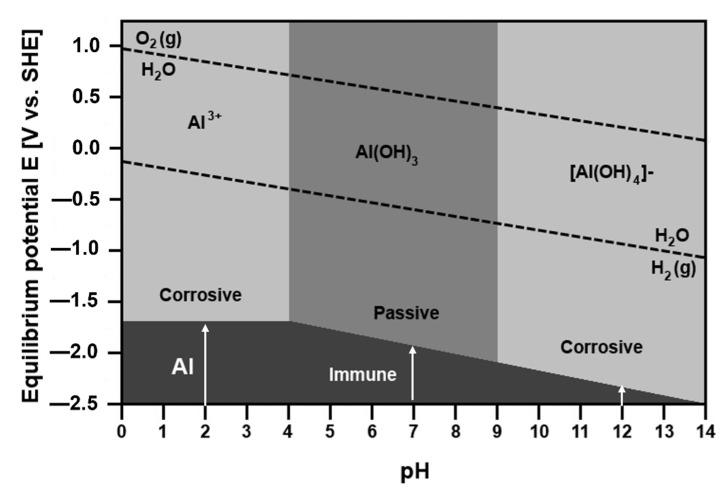
Equilibrium E–pH diagram of Al, replotted from [70].

Standard electrode potentials of metals are compared in Table 2. Since the Gibbs energy is related to electrode potential (Equation (3)), the tendency of a metal to corrode in given environment may be evaluated using E–pH plots. The diagrams have been calculated for most metals by Pourbaix and are available in ref. [69]. Figure 7 displays the E–pH diagram for Al–H_2_O system [69,70]. The plot indicates the stability regions of different phases in aqueous solutions. The E–pH diagram shows four different regions where metallic aluminum, aluminum cations (Al^3+^), aluminum hydroxide and complex anion [Al(OH)_4_]^−^ are stable. The region, where the metallic Al is stable, is labelled as immunity region. The areas with aluminum cations and anions as stable species are marked as corrosive regions. In these areas the corrosion occurs. The passivity region is where the solid hydroxide exists. In this region, Al is protected by a passive layer. The E–pH diagram demonstrates that corrosion takes place in both alkaline and acidic environments. The protective layer is formed at pH 4–9 [71]. The diagram also shows that the equilibrium electrode potential between [Al(OH)_4_]^−^ and Al, shifts to less noble values with increasing pH.

Although E–pH plots are useful in determining the metal’s tendency to corrode in the given environment, they do not provide a kinetic information. The rate of corrosion therefore needs to be determined separately by experimental methods. Corrosion rates are obtained either by weight loss measurements or electrochemical methods [67,68]. The weight loss measurement is a simple experiment to determine corrosion rates. In the experiment, a clean weighed piece of material with well-defined dimensions is exposed to the corrosive environment for a sufficient period. The corrosion rate (*v_corr_*) is then calculated based on the recorded weight loss according to the following equation [19,20]

(5) $$v_{corr}=\frac{\Delta w}{St}$$

In this equation, Δw is the weight loss, *t* is the reaction time and *S* is the exposed surface area.

Weight loss measurements, although useful, can be time-consuming and may not provide a complete information about reaction mechanism. Electrochemical techniques are therefore widely used to study the corrosion mechanisms of metals in different electrolytes. A potentiodynamic polarization is an electrochemical technique where the progress of reaction is controlled by potentiostat [67,68,70]. It brings in a variety of parameters and provides valuable information about reaction mechanism. In the experiment, three electrodes are assembled in a corrosion cell [72]. The corrosion cell includes a working electrode (sample), counter (auxiliary) electrode and reference electrode. During the experiment, the potential of the working electrode is systematically varied with respect to reference electrode. The resulting current is measured by counter electrode. The potential of reference electrode is constant and serves as reference value. Silver chloride (Ag/AgCl) and calomel electrodes (Hg/Hg_2_Cl_2_) immersed in a saturated KCl solution are most frequently used reference electrodes. Platinum mesh is used as counter electrode as this metal is corrosion resistant in most environments.

**Figure 8:**
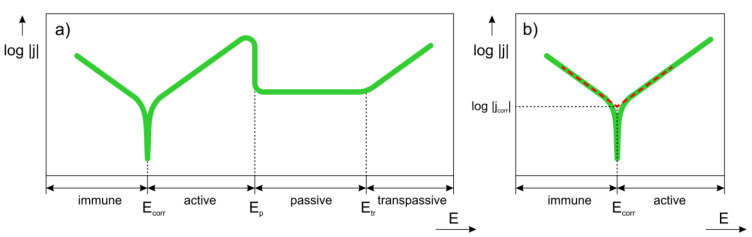
Schematic polarization curve of a passivating metal: (**a**) full curve, (**b**) Tafel extrapolation of cathodic and anodic regions.

A schematic potential versus current density curve recorded during the polarization experiment is given in Figure 8. Several different regions can be distinguished on the curve. The first region is immune region. In this region, observed at low potentials, the metal is thermodynamically stable. In immune region, cathodic reactions prevail at the metal surface. The second region is labelled as active region. It is observed once a corrosion potential, E_corr_, has been reached. In the active region, the metal actively corrodes according to Equation (2). The active corrosion means that the anodic dissolution of the metal takes place in the studied solution. Some metals can passivate. Therefore, a passivation region can be also observed on the polarization curve. The passive region corresponds to passive layer formation on the metal surface. The passivation is reflected by rapid current density increase or stabilization at potentials higher than E_p_ (passivation potential) on the polarization curve. At very high potentials, the current may start to abruptly increase. The increase is a result of passive film breakdown and happens at potential higher than transpassive potential, E_tr_. The passive film breakdown may be initiated by aggressive halide anions and lead to localized corrosion (pitting). A given alloy system may contain either some or all regions shown in Figure 8a.

The polarization curve provides a variety of electrochemical parameters. The corrosion potential and corrosion current density are important parameters that can be determined by Tafel extrapolation of the polarization curve. The procedure is shown in Figure 8b. In Tafel extrapolation, tangents to the polarization curve measured in immune and active regions are plotted. The intersection of the tangents determines the corrosion potential and corrosion current density. The corrosion potential reflects the tendency of the metal to corrode in given environment. The corrosion current corresponds to the rate of corrosion. In corrosion cell, a Faraday’s law is valid [67,68]. The weight loss of the metal at the working electrode, ∆*w*, is calculated by the following equation

(6) $$\Delta w=\frac{A}{zF}I_{corr}t$$

In this equation, *A* is the atomic weight of the metal, *I_corr_* is the corrosion current, *t* is the reaction time, *F* is Faraday’s constant and *z* is the number of electrons involved in the electrochemical reaction. The corrosion rate, *v_corr_*, is calculated from the corrosion current as

(7) $$v_{corr}=\frac{\Delta w}{St}=\frac{A}{zF}j_{corr}$$

In this equation *S* is the sample surface area and *j_corr_* is the corrosion current density (*j_corr_* = *I_corr_*/*S*). Equation (6) is valid for pure metals. For alloys, however, an equivalent weight, *E_w_* must be introduced to account for different molar masses of constituent metals and different valence states. The following equation defines the equivalent weight of an alloy [73]

(8) $$E_{w}=\frac{1}{\sum \frac{z_{i}f_{i}}{A_{i}}}$$

In the equation, *z_i_* is the valence state, *f_i_* is the weight fraction and *A_i_* is the atomic weight of metal *i* in the alloy. The corrosion rate than becomes

(9) $$v_{corr}=\frac{E_{w}}{F}j_{corr}$$

The assignment of valence states for TMs is often ambiguous as these elements have multiple stable valences. An independent experimental technique is therefore required, in addition to corrosion experiments, to establish the proper valence state. Another approach is to consult equilibrium Pourbaix diagrams [69]. The equilibrium E–pH diagrams can be used estimate the stable valence state of TM at the experimental conditions (electrode potential and pH of the electrolyte during corrosion test).

Metals become anodic and corrode only if their equilibrium half-cell potentials are smaller than the half-cell potential of the corresponding cathodic reaction [67,68]. When metals are combined into alloys it is no longer possible to define a unique half-cell potential. In multiphase alloys, different phases may act as local anodes and cathodes. The physical condition of the material may also be important. Constitutional variables such as the type and amount of structural defects (dislocations, grain boundaries) and crystal orientation are also important factors influencing the overall corrosion behavior.

Aluminum has a low standard electrode potential (Table 2, [68]). Therefore, aluminum and aluminum alloys are prone to corrosion. Nevertheless, the materials are also easily passivated. The passivation is related to spontaneous aluminum oxide/hydroxide film formation at the interface [74]. The passive film protects the material and impedes further reaction with the environment. Oxide layers grown on aluminum alloys at ambient temperatures are generally non-crystalline, although short-range cubic ordered structure has also been observed. In humid atmospheres, hydroxyl-oxides such as AlOOH or Al(OH)_3_ may also form on aluminum surface. The passive film is generally self-renewing and self-healing. Therefore, an accidental loss of the passive film due to, for example, abrasion is rapidly restored.

**Figure 9:**
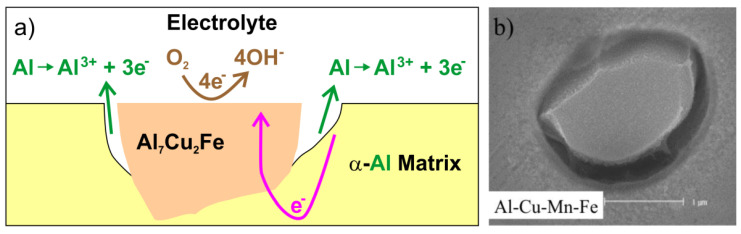
Schematic of the de-alloying and subsequent trenching of Al_2_CuMg intermetallic in AA2024 aluminum alloy (**a**); microstructure image of corroded alloy surface after 1-h exposure in aerated H_2_O at 30 °C (**b**), redrawn (**a**) and reproduced (**b**) from ref. [76].

Aluminum and its alloys are prone to pitting corrosion [75,76]. This type of local corrosion is often observed in seawater as it is initiated by chlorides and other halide anions in the electrolyte. The process may lead to passivity breakdown. Secondary phase particles are important constitutional variables affecting the corrosion rate. They can be classified into three different groups based on their electrochemical potential [76]: particles with active elements, noble elements, and particles with both active and noble elements. Reactive particles with active metals (such as Li, Mg or Zn) have low electrode potentials. These particles behave as anodes and subsequently dissolve when embedded in aluminum matrix. Particles with more noble elements (such as Fe or Cu) have higher electrode potentials and constitute local cathodes. They initiate anodic dissolution of Al matrix. The matrix adjacent to local cathode is preferentially attacked due to galvanic microcell created at the matrix/particle interface (Figure 9, [76]).

**Figure 10:**
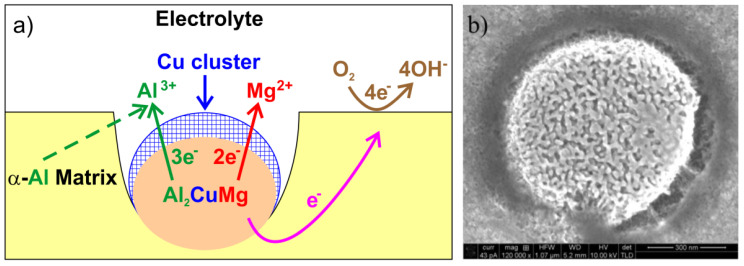
Schematic of trenching of Al_7_Cu_2_Fe intermetallic in AA2024-T3 aluminum alloy (**a**); microstructure image of corroded alloy surface after 1-h exposure in aerated H_2_O at 30 °C, redrawn (**a**) and reproduced (**b**) from ref. [76].

If intermetallic particles contain both noble and active elements, their electrochemical behavior changes over time. The active elements may preferentially dissolve, leaving behind the noble metals. This process is known as de–alloying. It is schematically shown in Figure 10 for Al_2_CuMg [76]. The galvanic interactions at the matrix-particle interface change because of de–alloying. The de–alloyed particle becomes nobler over time and may initiate an anodic dissolution of the surrounding matrix. Experimental conditions may also influence the particle dissolution behavior. For example, Al_20_Cu_2_Mn_3_ is a noble particle with respect to matrix at room temperature. Nevertheless, it may become anodic at temperatures higher than 50 °C. At 50 °C a dealloying behavior of Al_20_Cu_2_Mn_3_ has been observed, with de–alloying features much the same as Al_2_CuMg [77,78].

In this review we aim to address the following fundamental questions:

- Which SCIP of the alloy has the highest tendency to corrode?
- Which factors influence the positions of anodic and cathodic sites on the metal surface?
- Which factors affect the corrosion rate?
- In this paper the complex Al–TM alloys have been sorted according to their chemical composition.

## 4. Al–Co Alloys

**Figure 11:**
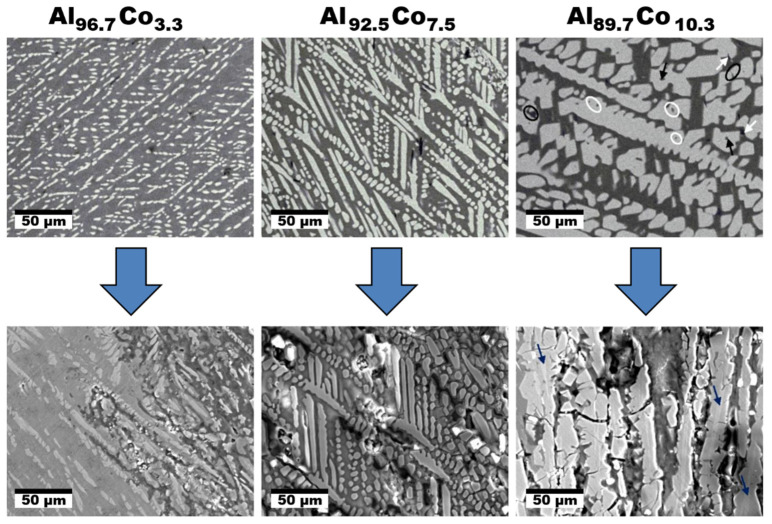
Microstructure of the rapidly solidified Al–Co alloys before and after corrosion in aqueous NaCl, adapted from reference [81].

The Al–Co alloys composed of SCIPs were investigated by Lekatou et al. [79,80,81,82] The authors prepared a series of novel Al–Co alloys with 3.3–10.3 at.% Co by arc-melting. The microstructures obtained were ranging from fully eutectic to hypereutectic microstructures with primary precipitation of structurally complex Al_9_Co_2_. Relatively uniform and directional microstructures were obtained (Figure 11, [81]). The fraction of directionally solidified Al_9_Co_2_ was increasing with increasing Co concentration. Microstructures of the materials before and after corrosion are compared in Figure 11. The alloys displayed a similar corrosion behavior in 3.5 wt.% NaCl. The corrosion attack resulted in a preferential dissolution of Al solid solution (ss).

A potentiodynamic polarization behavior of the Al–Co alloys in aqueous NaCl has been studied in refs. [81,82]. The Al_96.7_Co_3.3_ alloy showed a slightly superior corrosion performance compared to the remainder of the alloys as it had a relatively low corrosion current. Nevertheless, all Al–Co had a substantially higher corrosion resistance compared to Al. The anodic dissolution behavior was found to consist of four stages [81,82]. The individual stages were assigned to the following processes:

1. Active dissolution of Al(ss)
2. Passivation of Al(ss)
3. Breakdown of the passive film at the Al_9_Co_2_/Al(ss) interface and dissolution of the Al(ss) due to galvanic interaction with the nobler intermetallic (IMC)
4. Passivation of Al_9_Co_2_

At higher Co concentrations, a fragmentation of Al_9_Co_2_ in the corroded alloys occurred. The fragments piling in the gaps resulting from Al(ss) dissolution retarded the corrosion attack of the electrolyte. Al_9_Co_2_ had a higher electrochemical potential compared to Al(ss).

The corrosion behavior of Al–Co complex metallic alloys with 24–29 at.% Co was studied by Palcut et al. [83,84]. The following relative nobility of Al–Co CMAs has been found:

Al(ss) < Al_9_Co_2_ < Al_13_Co_4_ < Al_5_Co_2_ < β(AlCo) (10)

The nobility of IMCs increases with increasing Co concentration. The volume fractions of the phases and physical contacts between them play an important role in the alloy corrosion behavior. Results indicate that a galvanic mechanism is involved. Moreover, it should be mentioned that Al–Co IMCs are brittle [85]. Therefore, a piling of noble but brittle particles, such as β(AlCo), in pores resulting from massive dissolution of surrounding less-noble phases may significantly influence the alloy stability [82]. The structural defects in the alloy may act as rapid diffusion paths leading to a significant material degradation over time. The galvanic coupling of noble IMCs with more active phases may be critical to the alloy corrosion stability in halide-containing environments.

The parallel occurrence of SCIPs with similar chemical compositions has a positive effect on the corrosion susceptibility of the alloy [84,85]. The Al_74_Co_24_ alloy was composed of three phases with close chemical compositions (Z–Al_3_Co, Al_5_Co_2_, and Al_13_Co_4_, [84]). The Al_74_Co_24_ alloy had a higher corrosion potential compared to the remaining alloys which is an indicator of a superior corrosion resistance. The inspection of the alloy after corrosion testing revealed a relatively uniform phase dissolution [84]. The potential differences between constituent phases were probably small enough to initiate galvanic corrosion. The alloy corrosion could only be initiated at high electrode potentials. A polarization at high potentials resulted into a massive degradation of this alloy.

To further investigate the corrosion susceptibility of individual SCIPs with close chemical composition, an annealing of the Al_74_Co_26_ alloy at 1000 °C for 330 h has been carried out [86]. The annealing resulted in equilibrium microstructure of the alloy composed of Z–Al_3_Co and Al_5_Co_2_. The Z–Al_3_Co phase in the as-annealed Al_74_Co_26_ alloy was significantly less attacked. Although the bulk of this phase comprises less aluminum, it appears to be nobler and less susceptible to pitting corrosion compared to Al_5_Co_2_. The reason for this behavior could stem in a different structure of the phase surface. The Al_5_Co_2_ surface is terminated in puckered layers [87]. The surface of Z–Al_3_Co, on the other hand, is more densely populated compared to Al_5_Co_2_ [88]. Therefore, Z–Al_3_Co was less prone to corrosion attack.

**Figure 12:**
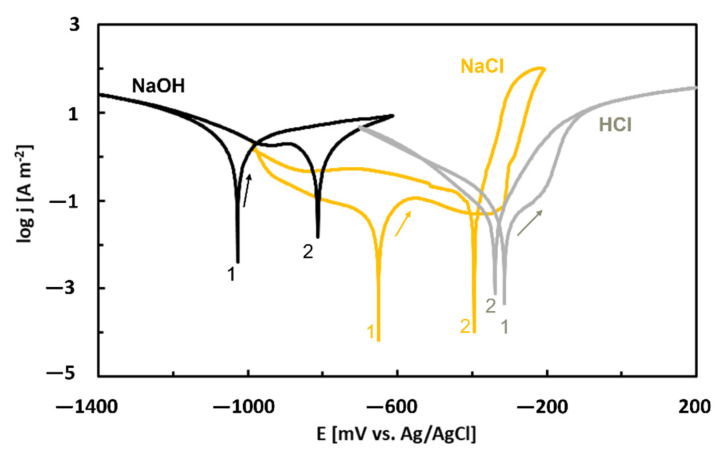
Potentiodynamic cyclic polarization curves of near-equilibrium Al_74_Co_26_ alloy in different electrolytes, re-plotted from reference [86].

The corrosion behavior of the as-annealed Al_74_Co_26_ alloy was investigated in neutral (NaCl, 0.6 mol dm^−3^), alkaline (NaOH, 10^−2^ mol dm^−3^) and acidic electrolytes (HCl, 10^−2^ mol dm^−3^) by cyclic potentiodynamic polarization [86]. The potentiodynamic curves are shown in Figure 12. Anodic parts of the curves measured in HCl and NaCl solutions displayed a passive region which was followed by an abrupt current density increase. When the polarization scan was reversed, a positive hysteresis was found. These features indicate pitting corrosion. The polarization behavior in NaOH, on the contrary, corresponds to uniform alloy corrosion.

The forward curves were evaluated by Tafel extrapolation and corrosion currents and corrosion potentials were obtained [86]. The lowest corrosion potential and highest corrosion current were found for NaOH. The highest corrosion potential and lowest corrosion current, on the other hand, were observed for the HCl solution. This behavior is in accordance with equilibrium E–pH diagram of Al (Figure 7).

## 5. Al–Cr Alloys

The Al–Cr alloys are expected to demonstrate a good corrosion resistance due to high concentrations of Al and Cr [89]. Both are passivating elements producing protective scales. The corrosion resistance of an Al_70_Cr_20_Fe_10_ alloy was studied by Li et al. [90]. The authors used commercial gas-atomized Al_70_Cr_20_Fe_10_ powders that were consolidated by spark plasma sintering. The phases present in the sintered Al–Cr–Fe pellets were the following: an icosahedral phase (i–Al–Cr–Fe), decagonal phase (d–Al–Cr–Fe) and crystalline Al_8_(Cr,Fe)_5_ and Al_9_(Cr,Fe)_4_ phases. Authors measured an open circuit potential (OCP) of the alloy in 3.5 wt. % NaCl and found that the OCP was nobler compared to Al. The OCP of the alloy was stable over time, indicating that an equilibrium has been rapidly established on the alloy surface. The Al_70_Cr_20_Fe_10_ alloy had a nobler corrosion potential and hence a lower susceptibility to corrosion compared to Al. It passivated in saline solution spontaneously due to significant amount of Cr. The alloy had a higher corrosion rate compared to pure Al [90]. Nevertheless, the corrosion rate was close to that of 316 stainless steel and smaller than AISI 440C stainless steel or AISI H13 tool steel.

**Figure 13:**
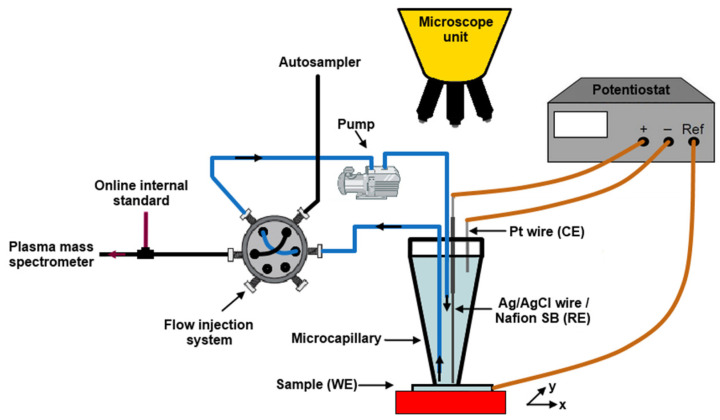
Schematic of the microcapillary flow ICP MS setup, re-drawn from reference [91].

The passivation behavior of Al–Cr–Fe alloys was studied by Ott et al. [91] The authors used a flow microcapillary plasma mass spectrometry. The schematic of the experimental set up is shown in Figure 13 [91]. In the experiment, a tiny microcapillary was positioned on the alloy surface and continuously filled with the desired solution. The flow injection was operated in loops by switching the valve. The loop volume was continuously transferred to the inductively coupled plasma mass spectrometer (ICP MC) for element analysis. The microcapillary was refilled with fresh electrolyte from the reservoir. The circulation was ensured by a peristaltic pump. A microscope was included to control precise positioning of the capillary on the alloy surface. The technique provided time-resolved information about transient electrochemical processes and element-specific dissolution at the metal–electrolyte interface.

**Figure 14:**
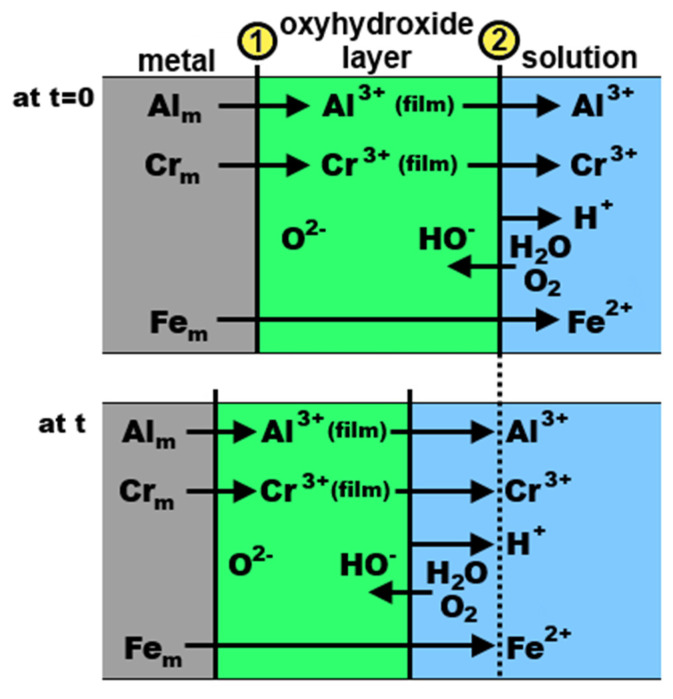
Schematic of the passive film evolution on γ-phase Al_64.2_Cr_27.2_Fe_8.1_ alloy, re-drawn from reference [91].

The authors prepared and studied a polycrystalline γ-phase Al_64.2_Cr_27.2_Fe_8.1_ alloy (composition given in at.%, [91]). The corrosion behavior was studied in two acidic solutions: H_2_SO_4_ (pH 0) and HCl (pH 2). In sulfuric acid, very low element dissolution rates were found. Neither Fe nor Al is stable at low pH [69]. Therefore, Cr is an essential element in the passive film stability. It helps to stabilize the Al cations within the passive film, as evidenced by a low Al release over 2 h. A possibly mixed oxy-hydroxide of Al and Cr was suggested to have been formed on the alloy surface (Figure 14, [91]). The dynamic passivation mechanism is related to the fact that the cation dissolution occurring at the oxyhydroxide–solution interface (②) is compensated by additional film growth at the metal–oxyhydroxide interface (①). Longer air-aging was found to be beneficial for stabilizing the passive film.

In chloride-containing hydrochloric acid, ten times higher Al dissolution rates were found at the OCP, suggesting a decreasing stability of the spontaneously formed passive film [91]. The thickness of the dissolved passive film was much higher compared to H_2_SO_4_ (Table 3). But even in HCl, a potentiostatic polarization at 0.18V_SCE_ slowed down the dissolution processes at the oxyhydroxide–solution interface by a factor of 6. The electrochemical polarization at low passive potentials induces electrical field generated oxide film modification, thereby increasing the chemical stability at the oxyhydroxide–solution interface. In the high potential passive region, a localized attack was initiated with subsequent active metal dissolution.

**Figure 15:**
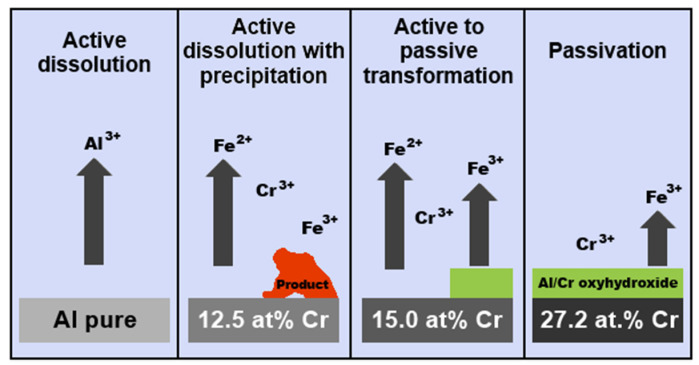
Schematic of passivation/dissolution processes on pure Al and Al–Cr–Fe alloys in aqueous HCl + NaCl mixture with initial pH 2, re-drawn from reference [92].

The passivation behavior of Al–Cr–Fe complex metallic alloys in NaCl+HCl mixtures was further investigated by Beni et al. [92] The authors prepared three alloys: polycrystalline single phase Al_79.5_Cr_12.5_Fe_8.0_ (composed of orthorhombic phase), Al_64.2_Cr_27.2_Fe_8.1_ (single phase, composed of cubic γ phase) and single crystalline orthorhombic Al_79.0_Cr_15.0_Fe_6.0_. The corrosion behavior of the different alloys could be explained considering the passivating role of Cr combined with Fe oxyhydroxide precipitation. The anticipated reaction mechanism is presented in Figure 15 [92]. The Al_79.5_Cr_12.5_Fe_8.0_ alloy was found to undergo an active dissolution in the electrolyte, as proven by the high element concentrations in solution measured by ICP MS. The chromium concentration (12.5 at.%) was small but sufficient to stabilize the initially air-formed oxyhydroxide for 22 days, as evidenced by the constant low pH of the solution and low dissolution compared to Al. The concentration of Cr was, however, too low to provide a long-term protection. A thick and non-protective layer has been formed on the surface. With increasing Cr concentration, a protective layer on the alloys started to form. The Cr concentration of 15.0 at.% was sufficient to stabilize the passive film up to 78 days. A complete and long-lasting protective scale was finally achieved at 27.2 at.% Cr [92,93].

## 6. Al–Noble-Metal Alloys

Massiani et al. [35] investigated the corrosion behavior of crystalline and quasicrystalline phases in the Al–Cu–Fe(–Cr) alloys by potentiodynamic polarization in strongly acidic and alkaline solutions. They found that the corrosion resistance was determined by the alloy chemical composition. The complex crystal structure had only a minor influence. Rüdiger and Köster [94] found that the corrosion behavior of quasicrystals and their approximants in the Al–Cu–Fe alloy system could be explained based on the electrochemical behavior of the component elements. The surface of the icosahedral Al_63_Cu_25_Fe_12_ was covered by a thick non-protective layer composed of Cu_2_O, Al(OH)_3_ and metallic Cu. The scale chemical composition was comparable to crystalline Al_7_Cu_2_Fe. The complex crystal structure thus did not have a substantial influence on the corrosion resistance [94]. Furthermore, the authors observed a formation of porous Cu layer in i–Al_63_Cu_25_Fe_12_ phase at pH 0.

While Rüdiger and Köster studied single phase quasicrystalline alloys, Huttunen et al. investigated Al–Cu–Fe alloys composed of several different phases (Table 4, [95]). The corrosion behavior was determined by anodic polarization. The microstructural features and phase constitution of the alloys before and after the polarization were studied by scanning electron microscopy and X-ray diffraction.

The study was focused on four different Al–Cu–Fe alloys: Al_67.5_Cu_20_Fe_12.5_, Al_65_Cu_20_Fe_15_, Al_62.5_Cu_25_Fe_12.5_ and Al_60_Cu_27.5_Fe_12.5_ [95]. The authors found that the presence of structurally complex phases did not improve the alloys corrosion resistance [95,96]. The chemical composition of the phases, however, was of great importance. The corrosion potentials of Al–Cu–Fe alloys with Cu-rich phases were nobler and had lower corrosion rates compared to Cu-lean alloys [95]. Relative amounts of the phases and their electrical contacts were also significant factors influencing the overall corrosion behavior. Phases with high Cu concentration remained virtually unaffected by corrosion. The phases with low Cu atomic fractions were susceptible to corrosion attack. This behavior could be explained by the higher electrode potential of Cu compared to Al and Fe (Table 2). The corrosion was found to occur by galvanic mechanism near phase boundaries. The corrosion behavior of Al–Cu–Fe alloys was studied in alkaline, neutral, and acidic solutions. In alkaline and neutral electrolytes, an oxidation of Al and Cr occurred on the surface of the alloys. The oxidation was accompanied with Cu deposition on the alloy surface. The Cu deposition interfered with passive layer formation and introduced pores into the oxide film. The icosahedral ψ–Al_65_Cu_20_Fe_15_ was the only phase capable of forming a stable passive layer on the surface [95].

The Al–noble-metal alloys are interesting materials from electrochemical point of view. The alloys are prone to selective dissolution of less noble elements (leaching) because of markedly different electrode potentials of the constituent metals [97]. The less noble elements tend to dissolve in the electrolyte, leaving behind their vacant positions. As a result of leaching, a porous de-alloyed structure forms on the alloy surface [97]. The leaching can be either uniform or localized. Examples of leaching include preferential dissolution of Zn from brass (de-zincification) or Fe removal from gray cast iron (a so-called graphitic corrosion) [98]. Other examples include de-aluminification, de-nickelification and de-cobaltification [99].

**Figure 16:**
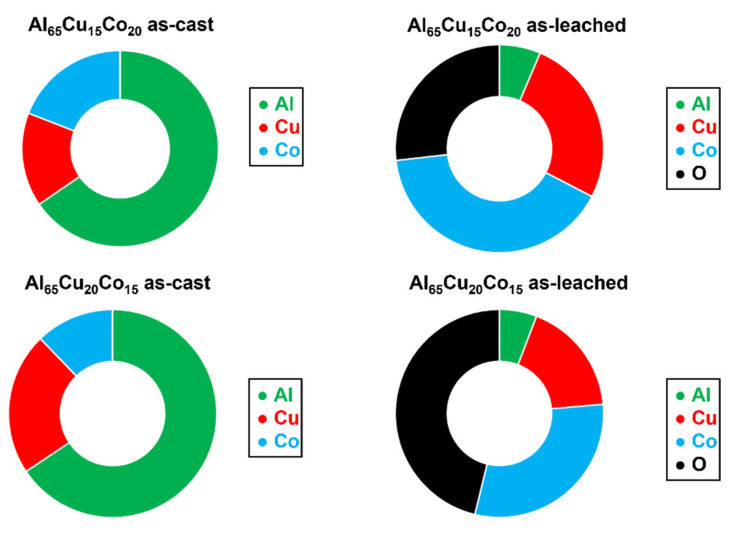
Chemical composition (in at.%) of as-cast and as-leached Al–Cu–Co alloys, plotted from data in reference [100].

Mishra et al. studied a chemical leaching of Al–Cu–Co decagonal quasicrystals [100]. The authors prepared two alloys with Al_65_Cu_15_Co_20_ and Al_65_Cu_20_Co_15_ chemical compositions and studied their corrosion behavior in aqueous NaOH (10 mol L^−1^). The alloys were immersed in the alkaline solution at room temperature for 8 h. Most Al atoms were removed (Figure 16, [100]). A nearly uniformly distributed metallic Cu, Co and Co_3_O_4_ nanoparticles were found on the alloy surface after leaching. The crystallite size was calculated from the XRD reflections’ broadening and further confirmed by TEM [100]. The nanostructure formation of the leached layer was controlled by Al dissolution rate during leaching. The dispersed Cu and Co nanoparticles were stable in the leached layer and Cu agglomeration was suppressed.

Porous nanostructures composed of noble metals are important catalysts. The formation of Cu-rich porous nanostructure from decagonal Al_65_Co_x_Cu_35−x_ alloys (x = 12.5, 15, 17.5 at.%) was studied by Kalai Vani et al. [101] A selective dissolution of Al and Co was achieved by combined immersion of the alloys in both NaOH (5 mol L^−1^) and Na_2_CO_3_ (0.5 mol L^−1^) electrolytes. A high specific surface of 30 m^2^ g^−1^ of the porous Cu structure was achieved.

The electrochemical de-alloying of binary Al–noble metal alloys was also studied [102]. It has been shown that nano-porous Pd, Ag and Au with various structures can be produced through electrochemical leaching of the Al–based alloys in NaCl aqueous solution. Galvanic interactions between coexisting phases dominate during corrosion of double phase alloys. The level of de-alloying depends on the critical de–alloying potential [103], diffusion of the noble element and reactivity of the noble element and chloride anion. The porosity evolution is a dynamic process. It is not a simple excavation of the less noble phase from two phase material. The formation of the porous nanostructure involves selective leaching of Al and is accompanied with coarsening of the noble element due to surface diffusion.

**Figure 17:**
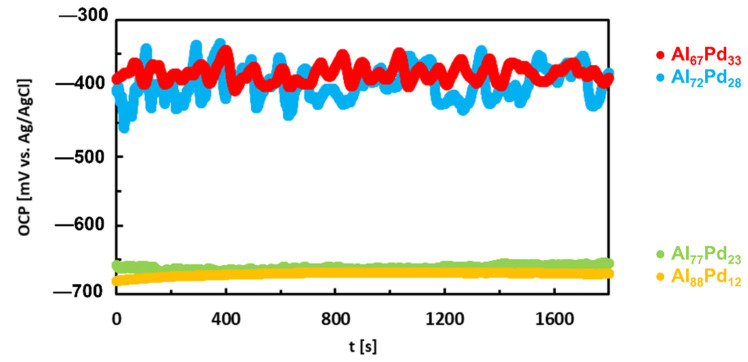
Open circuit potentials of Al–Pd alloys in aqueous NaCl, re-plotted from data in reference [105].

The corrosion behavior of Al–Pd alloys composed of SCIPs was studied in references [104,105]. The open circuit potentials are given in Figure 17. The OCPs decrease in the following order:

Al_67_Pd_33_, Al_72_Pd_28_ (group I) > Al_77_Pd_23_, Al_88_Pd_12_ (group II) (11)

The OCPs of the alloys decrease with decreasing Pd concentration. This observation is in accordance with expectations since Al is electrochemically more active compared to Pd (Table 2). The corrosion resistance of both as-annealed and as-solidified alloys was comparable. A large difference, however, between OCP and E_corr_ has been found for group I alloys (Al_67_Pd_33_ and Al_72_Pd_28_). The OCPs of these alloys were comparable to their pitting potentials obtained by potentiodynamic polarization. The Al_67_Pd_33_ and Al_72_Pd_28_ alloys were probably in a pitting corrosion stage during the OCP measurement. This suggestion was manifested by oscillations of OCP resulting from a possible pitting behavior (Figure 17). The anodic dissolution of the alloy at pits requires a generation of cathodic current from the surrounding surface. The electric current bursts are transient and cause a temporary decrease in the OCP value. The pitting corrosion sites are usually very small. However, the current densities during transient bursts inside the pits can be up to 1 A/m^2^ [106]. The significant corrosion rates of the alloy are due to aggressive environments developed inside the pits. Although the pits are small, they may affect the electrochemical response of much larger surface areas. Therefore, the differences in current densities on separated anodic and cathodic sites are reflected in potential oscillations (so-called electrochemical noise associated with localized corrosion).

Interactions between phases with different chemical composition play a significant role in alloy corrosion. The Al_67_Pd_33_ and Al_72_Pd_28_ alloys were found to be composed of structurally complex ε_n_ (Al_3_Pd) and δ(Al_3_Pd_2_). The electrochemical nobility of Al–Pd phases in aqueous NaCl (0.6 mol L^−1^) increases in the following order

(Al) < ε_n_(Al_3_Pd) < δ(Al_3_Pd_2_) (12)

The δ phase has a higher concentration of Pd. It serves as a cathode, and thereby further accelerates the anodic dissolution of the surrounding ε_n_ phase. The corrosion mechanism of Al–Pd alloys in aqueous NaCl involves a rapid passivation stage on the alloy surface [105,106]. However, once a breakdown potential is reached during potentiodynamic polarization, the passive layer becomes unstable and susceptible to local attack by chloride anions. Consequently, chloro–aluminum complex cations are formed and released into the solution. The local disruption of the passive layer reveals a naked alloy surface which becomes more susceptible to further corrosion attack.

**Figure 18:**
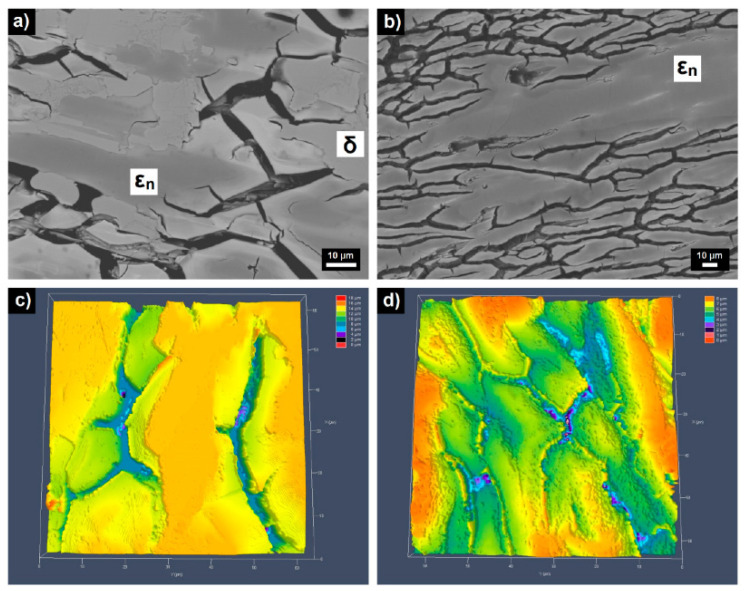
Microstructure of as-corroded Al–Pd–Co alloys: backscatter scanning electron microscopy images of Al_70_Pd_25_Co_5_ (**a**) and Al_74_Pd_12_Co_14_ (**b**) and confocal laser scanning images of Al_70_Pd_25_Co_5_ (**c**) and Al_74_Pd_12_Co_14_ (**d**). Reproduced from reference [107].

The microstructures of as-annealed and as-solidified Al_72_Pd_28_ and Al_67_Pd_33_ alloys had similar features after corrosion testing. In the alloys a high number of inter-penetrating channels have been found [105,106]. Pits were also observed in the inter-connection between the channels. The formation of channels was driven by pitting and de–alloying. The pits were probably initiation sites of the channels. A preferential de-alloying of Al (de-aluminification) has also been observed. The preferential leaching of Al led to initiation of microcracks. During rapid solidification residual stresses have been accumulated in the alloys. The stresses were released during leaching, resulting in continuous tunnels inter-penetrating the surfaces of de-alloyed materials. A similar corrosion behavior was also found for the Al–Pd–Co alloys (Figure 18, [107]). The de-alloying of Al was more pronounced in the as-solidified alloys. This is probably a consequence of their higher defect concentrations compared to as-annealed alloys. The de–alloying behavior was significantly reduced in as-annealed alloys [105].

## 7. Comparison of Al–TM Alloys with Different Chemical Composition

**Figure 19:**
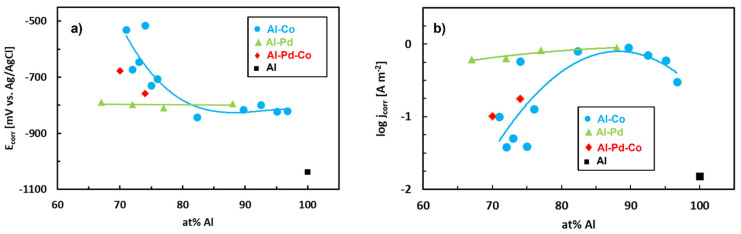
Corrosion parameters of Al–Pd, Al–Co and Al–Pd–Co alloys in aqueous NaCl (0.6 mol L^−1^). Data is compiled from references [81,82,84,105,107].

Corrosion parameters of previously studied Al–based CMAs are given in Table 5. The corrosion behavior of Al–Co, Al–Pd and Al–Pd–Co alloys in aqueous NaCl (0.6 mol L^−1^) is compared in Figure 19. Corrosion potentials of Al–Co alloys decrease with increasing Al concentration. The Al–Pd alloys have lower corrosion potentials. The values are smaller than the corrosion potentials of the remaining two alloy groups. Furthermore, the corrosion currents of Al–Pd alloys are higher compared to the Al–Pd–Co and Al–Co alloys (Figure 19b). These observations indicate that Al–Pd alloys are more susceptible to corrosion attack compared to the remaining two alloy groups.

The corrosion behavior of the Al–Pd–Co alloys is closer to Al–Co alloys (Figure 19). This observation is unexpected, since Al–Co–Pd and Al–Co alloys have different phase constitutions. Moreover, the preferentially corroding phase is ε_n_ in the Al–Pd–Co alloys. ε_n_ is absent in the Al–Co alloys. It can be noted that Co substitution for Pd significantly improves the corrosion resistance of ε_n_. The positive influence of Co on the corrosion resistance of Al–TM alloys has also been observed by Sukhova and Polonskyy [108]. It is therefore the chemical composition and not the crystal structure of the phase that plays a dominant role in the corrosion resistance. 

**Figure 20:**
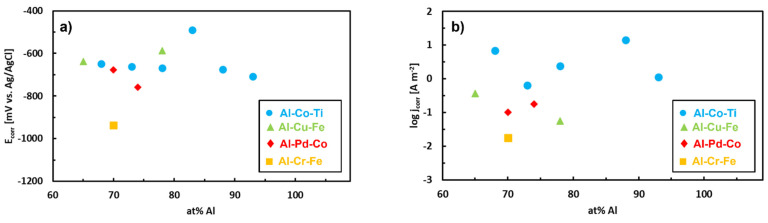
Corrosion parameters of ternary Al–TM alloys in aqueous NaCl (0.6 mol L^−1^). Data is compiled from references [90,107,109,110].

To further probe the role of chemical composition, we have compared the corrosion parameters of the previously discussed Al–TM alloys. The data compilation is plotted in Figure 20. The parameters are relatively scattered due to large differences in the overall alloy chemical compositions (Table 5). Nevertheless, some general trends can be noted. The as-solidified Al–Pd–Co alloys have corrosion current densities comparable to Al–Cu–Fe alloys. The corrosion potentials of the Al–Pd–Co and Al–Cu–Fe alloys are close to −650 mV (vs. Ag/AgCl)). The Al–Cr–Fe alloy is also included in Figure 20. This alloy has a lower corrosion potential compared to the remainder of the alloys. This is related to the absence of noble metals, such as Pd, in the alloy. Furthermore, the Al–Cr–Fe alloy has a low corrosion current due to the presence of Cr [90]. This element is responsible for a rapid passive layer formation on the alloy surface.

The corrosion parameters of Al–Co–Ti alloys [110] are also included in the same figure. The corrosion potentials of these alloys are comparable to Al–Pd–Co alloys (Figure 20). The concentration of Ti in the Al–Co–Ti alloys was constant (2 at.%). The atomic fraction of Co was varied between 5–30 at.%. Due to small and constant Ti concentration, the microstructural features of the Al–Co–Ti alloys were comparable to Al–Co alloys [81,84]. The corrosion current densities of Al–Pd–Co alloys, however, were smaller compared to Al–Co–Ti alloys. The Al–15Co–2Ti alloy was an exception as the alloy demonstrated a lower corrosion current compared to the remainder of the alloys. The difference was related to different intermetallic particles contained in the alloy (Al_13_Co_4_, Al_9_Co_2_, and Al_3_Ti). They had different volume fractions and morphologies compared to the remaining Al–Ti–Co alloys [110]. The observations show that specific Co atomic fractions may significantly increase the corrosion resistance of the bulk Al–TM alloys. The ε_n_ phase of the Al–Pd–Co alloys had a high concentration of Co. The Co additions significantly contributed to the superior corrosion performance of the bi-phasic Al_70_Pd_25_Co_5_ alloy.

The corrosion parameters of the structurally complex Al–TM phases are comparable to previously studied Al–TM intermetallic phases with simpler structures [111]. Therefore, it is the chemical composition of the SCIP and not the crystal structure that influences the corrosion behavior. The electrochemical activity of the SCIPs may also vary with time. Zhu et al. investigated the corrosion performance of Al–TM intermetallic phases over time [78]. At early stages of exposure, a de–alloying was the primary corrosion mechanism. The de–alloying led to an ennoblement of intermetallic particles over time due to preferential Al leaching [78]. The ennoblement speeded up an anodic dissolution of the adjacent matrix and worsened the corrosion behavior. A long-term annealing may also influence the corrosion performance of the alloy constituent phases. It reduces internal stresses generated during casting and contributes to a more uniform element redistribution in the SCIPs.

## 8. Conclusions

In this paper the electrochemical corrosion behavior of Al–TM alloys composed of SCIPs has been reviewed. The following conclusions can be drawn: 

1. The Al–TM alloys have a capability of forming passive layers because of high Al concentration. The Al–Cr alloys, for example, can form protective passive layers of considerable thickness in different electrolytes.

2. In halide-containing solutions the Al–TM alloys are prone to pitting corrosion. Galvanic microcells between different SCIPs form which may further accelerate the localized corrosion attack.

3. The electrochemical activity of aluminum–transition-metal SCIPs is primarily determined by electrode potential of the alloying element(s). The electrochemical nobility of individual SCIPs increases with increasing concentration of noble elements. The SCIPs with less noble elements tend to dissolve in contact with nobler particles. The SCIPs with noble metals are prone to selective de-alloying (de-aluminification). The electrochemical activity of SCIPs may change over time.

4. The chemical composition of the SCIPs has a primary influence on their corrosion properties. The structural complexity is secondary. It becomes important when phases with similar chemical composition come into close physical contact. The phase with higher structural complexity tends to be cathodic and can be retained during corrosion.

## Figures and Tables

**Table 1:** Examples of quasicrystalline phases, compiled from references [7,8,10,11,12,13,14,15,16,17,18,19,20,21,22,23].

Quasiperiodicity	Alloy Systems
one-dimensional quasiperiodic arrangement	Al–Ni–Si, Al–Cu–Co, Al–Cu–Mn, Mo–V
two-dimensional octagonal quasiperiodic arrangement	Cr–Ni–Si, V–Ni–Si, Mn–Si–Al, Mn–Si, Mn–Fe–Si
two-dimensional decagonal quasiperiodic arrangement	Al–Pd, Al–Ir, Al–Os, Al–Pt, Al–Rh, Al–Ru, Al–Fe, Al–Mn, Al–Ni, Al–Ni(Si), Al–Cr(Si), Al–Co, Al–Cu–Mn, Al–Mn–Fe, Al–Cu–Ni, Al–Cu–Co, Al–Co–Ni, V–Ni–Si, Al–Pd–Mn, Al–Pd–Co, Al–Pd–Fe, Al–Pd–Cr, Al–Pd–Os, Al–Pd–Ru, Ga–Fe-Cu–Si, Al–Mn–Fe-Ge, Zn–Ge–Dy
two-dimensional dodecagonal quasiperiodic arrangement	V–Ni, Cr–Ni, V–Ni–Si
three-dimensional icosahedral quasiperiodic arrangement	Al–Fe, Al–Mn, Al–Re, Al–Ru, Al–W, Al–Mo, Ti–Ni, Al–Cr–Ru, Mn–Ni–Si, Ni–Nb, Al–Cu–Mn, Al–Cu–Fe, Al–Pd–Mn, Zn–Mg–Y

**Table 2:** Standard potentials, E^0^, for metal electrodes, compiled from reference [68].

Electrode	E^0^[V_SHE_] ^a^	Electrode	E^0^[V_SHE_]	Electrode	E^0^[V_SHE_]
Au/Au^3+^	+1.498	Ni/Ni^2+^	−0.250	Zn/Zn^2+^	−0.763
Pt/Pt^2+^	+1.200	Co/Co^2+^	−0.277	Ti/Ti^2+^	−1.630
Pd/Pd^2+^	+0.978	Cd/Cd^2+^	−0.403	Al/Al^3+^	−1.662
Ag/Ag^+^	+0.799	Fe/Fe^2+^	−0.440	Mg/Mg^2+^	−2.363
Cu/Cu^2+^	+0.337	Cr/Cr^3+^	−0.744	Li/Li^+^	−3.045

^a^ Volts versus standard hydrogen electrode.

**Table 3:** Estimated equivalent passive film thickness [91].

Electrolyte	pH	Ageing Time[h]	Applied Potential [V_SCE_] ^a^	Dissolved Passive Film Thickness [nm]	Formed Passive Film Thickness [nm]
H_2_SO_4_	0	0.5	OCP ^b^	106	
H_2_SO_4_	0	0.5	0.18	55.6	8.4
H_2_SO_4_	0	0.5	0.68	153	6.9
H_2_SO_4_	0	3	OCP	116	
HCl	2	0.5	OCP	880	
HCl	2	0.5	0.18	147	24.6

^a^ Volts versus saturated calomel electrode, ^b^ Open circuit potential.

**Table 4:** Chemical composition of SCIPs in Al–Cu–Fe alloys (in at.%) studied in reference [95].

Alloy	θ–Al_2_Cu			ψ–Al_65_Cu_20_Fe_15_			β–AlFe			λ–Al_13_Fe_4_		
	Al	Cu	Fe	Al	Cu	Fe	Al	Cu	Fe	Al	Cu	Fe
**Al_60_Cu_27.5_Fe_12.5_**	49.8	49.5	0.7	65.2	22.5	12.3	67.6	32.2	0.2	73.4	4.5	22.1
**Al_62.5_Cu_25_Fe_12.5_**	47.2	52.3	0.5	62.0	27.4	10.6	65.7	33.9	0.4	71.8	7.5	20.7
**Al_65_Cu_20_Fe_15_**	51.5	47.7	0.8	65.3	22.9	11.8	68.3	31.4	0.3	73.7	4.3	22.0
**Al_67.5_Cu_20_Fe_12.5_**	66.4	33.0	0.6	70.3	20.1	9.6	93.8	5.8	0.4	73.7	3.4	22.9

**Table 5:** Electrochemical corrosion parameters of Al-based complex metallic alloys.

Alloy	Condition	Electrolyte	E_corr_ [mV vs. Ag/AgCl]	j_corr_ [A m^−2^]	Reference
Al_96.7_Co_3.3_	Cast	Aerated NaCl (0.6 mol dm^−3^)	−838 ± 20	0.7 ± 0.1	[79]
Al_96.7_Co_3.3_	Arc-melted	Aerated NaCl (0.6 mol dm^−3^)	−820 ± 36	0.3 ± 0.1	[79]
Al_96.7_Co_3.3_	Powder-metallurgy sintered	Aerated NaCl (0.6 mol dm^−3^)	−890 ± 50	0.9 ± 0.2	[79]
Al_96.7_Co_3.3_	Cast	Aerated NaCl (0.6 mol dm^−3^)	−820 ± 36	0.3 ± 0.1	[81]
Al_95.1_Co_4.9_	Cast	Aerated NaCl (0.6 mol dm^−3^)	−823 ± 23	0.6 ± 0.1	[81]
Al_92.5_Co_7.5_	Cast	Aerated NaCl (0.6 mol dm^−3^)	−799 ± 23	0.7 ± 0.1	[81]
Al_89.7_Co_10.3_	Cast	Aerated NaCl (0.6 mol dm^−3^)	−816 ± 23	0.9 ± 0.1	[81]
Al_82.3_Co_17.7_	Cast	Aerated NaCl (0.6 mol dm^−3^)	−843 ± 16	1.6 ± 0.1	[82]
Al_82.3_Co_17.7_	Arc-melted	Aerated NaCl (0.6 mol dm^−3^)	−825 ± 18	0.8 ± 0.1	[82]
Al_82.3_Co_17.7_	Powder-metallurgy sintered	Aerated NaCl (0.6 mol dm^−3^)	−877 ± 23	5.8 ± 0.6	[82]
Al_99.1_Co_0.9_	Arc-melted	Aerated H_2_SO_4_ (1 mol dm^−3^)	−400 ± 7	1.9 ± 0.3	[80]
Al_97.6_Co_2.4_	Arc-melted	Aerated H_2_SO_4_ (1 mol dm^−3^)	−406 ± 2	3.6 ± 0.6	[80]
Al_96.7_Co_3.3_	Arc-melted	Aerated H_2_SO_4_ (1 mol dm^−3^)	−388 ± 10	2.6 ± 0.6	[80]
Al_95.1_Co_4.9_	Arc-melted	Aerated H_2_SO_4_ (1 mol dm^−3^)	−390 ± 5	1.9 ± 0.6	[80]
Al_92.5_Co_7.5_	Arc-melted	Aerated H_2_SO_4_ (1 mol dm^−3^)	−381 ± 18	3.1 ± 0.8	[80]
Al_89.3_Co_10.3_	Arc-melted	Aerated H_2_SO_4_ (1 mol dm^−3^)	−372 ± 7	2.9 ± 0.4	[80]
Al_76_Co_24_	Cast	Aerated NaCl (0.6 mol dm^−3^)	−706	0.13	[84]
Al_75_Co_25_	Cast	Aerated NaCl (0.6 mol dm^−3^)	−729	0.039	[84]
Al_74_Co_26_	Cast	Aerated NaCl (0.6 mol dm^−3^)	−515	0.58	[84]
Al_73_Co_27_	Cast	Aerated NaCl (0.6 mol dm^−3^)	−646	0.05	[84]
Al_72_Co_28_	Cast	Aerated NaCl (0.6 mol dm^−3^)	−672	0.04	[84]
Al_71_Co_29_	Cast	Aerated NaCl (0.6 mol dm^−3^)	−530	0.10	[83]
Al_74_Co_26_	Annealed in Ar 1050 °C 330 h	Aerated NaCl (0.6 mol dm^−3^)	−651	0.051	[86]
Al_74_Co_26_	Annealed in Ar 1050 °C 330 h	Aerated HCl (0.01 mol dm^−3^)	−314	0.032	[86]
Al_74_Co_26_	Annealed in Ar 1050 °C 330 h	Aerated NaOH (0.01 mol dm^−3^)	−1026	2.6	[86]
Al_72_Fe_15_Ni_13_	Cast	Aerated NaCl (0.87 mol dm^−3^)	-	1.4	[108]
Al_69_Co_21_Ni_10_	Cast	Aerated NaCl (0.87 mol dm^−3^)	-	1.2	[108]
Al_70_Cr_20_Fe_10_	Powder metallurgy sintered	Aerated NaCl (0.6 mol dm^−3^)	−938	0.018	[90]
Al_65_Cu_20_Fe_15_	Cast	NaCl (0.6 mol dm^−3^)	−638 ± 100	0.37	[109]
Al_78_Cu_7_Fe_15_	Cast	NaCl (0.6 mol dm^−3^)	−586 ± 100	0.056	[109]
Al_80_Cu_5_Fe_14_Si_1_	Cast	NaCl (0.6 mol dm^−3^)	−570 ± 100	0.14	[109]
Al_70_Cu_9_Fe_10.5_Cr_10.5_	Cast	Na_2_SO_4_ (0.5 mol dm^−3^)	−556	1.6 × 10^−2^	[35]
Al_64_Cu_24_Fe_12_	Cast	Na_2_SO_4_ 0.5 mol dm^−3^)	−555	7.3 × 10^−2^	[35]
Al_63_Cu_20_Co_15_Si_2_	Cast	Na_2_SO_4_ (0.5 mol dm^−3^)	−635	2.2 × 10^−2^	[35]
Al_70_Cu_9_Fe_10.5_Cr_10.5_	Cast	Na_2_SO_4_ (0.5 mol dm^−3^) + H_2_SO_4_ (pH 2)	−496	1.4 × 10^−2^	[35]
Al_64_Cu_24_Fe_12_	Cast	Na_2_SO_4_ (0.5 mol dm^−3^) + H_2_SO_4_ (pH 2)	−512	0.8 × 10^−2^	[35]
Al_63_Cu_20_Co_15_Si_2_	Cast	Na_2_SO_4_ (0.5 mol dm^−3^) + H_2_SO_4_ (pH 2)	−186	0.6 × 10^−2^	[35]
Al_70_Cu_9_Fe_10.5_Cr_10.5_	Cast	NaOH (0.1 mol dm^−3^)	−921	1.6 × 10^−2^	[35]
Al_64_Cu_24_Fe_12_	Cast	NaOH (0.1 mol dm^−3^)	−1508	336 × 10^−2^	[35]
Al_63_Cu_20_Co_15_Si_2_	Cast	NaOH (0.1 mol dm^−3^)	−1441	462 × 10^−2^	[35]
Al_72_Pd_20_Mn_8_	Annealed in Ar 800 °C 12 h	Deaerated NaCl (0.5 mol dm^−3^)	−355	0.5	[36]
Al_88_Pd_12_	Cast	Aerated NaCl (0.6 mol dm^−3^)	−794	0.89	[105]
Al_77_Pd_23_	Cast	Aerated NaCl (0.6 mol dm^−3^)	−809	0.82	[105]
Al_72_Pd_28_	Cast	Aerated NaCl (0.6 mol dm^−3^)	−797	0.63	[105]
Al_67_Pd_33_	Cast	Aerated NaCl (0.6 mol dm^−3^)	−798	0.62	[105]
Al_77_Pd_23_	Annealed in Ar 700 °C 500 h	Aerated NaCl (0.6 mol dm^−3^)	−763	0.75	[105]
Al_72_Pd_28_	Annealed in Ar 700 °C 500 h	Aerated NaCl (0.6 mol dm^−3^)	−841	0.68	[105]
Al_67_Pd_33_	Annealed in Ar 700 °C 500 h	Aerated NaCl (0.6 mol dm^−3^)	−783	0.72	[105]
Al_88_Pd_12_	Cast	Aerated HCl (0.01 mol dm^−3^)	−478	0.26	[104]
Al_77_Pd_23_	Cast	Aerated HCl (0.01 mol dm^−3^)	−450	0.27	[104]
Al_72_Pd_28_	Cast	Aerated HCl (0.01 mol dm^−3^)	−253	0.03	[104]
Al_67_Pd_33_	Cast	Aerated HCl (0.01 mol dm^−3^)	−200	0.17	[104]
Al_88_Pd_12_	Cast	Aerated NaOH (0.01 mol dm^−3^)	−1019	0.42	[104]
Al_77_Pd_23_	Cast	Aerated NaOH (0.01 mol dm^−3^)	−1033	0.25	[104]
Al_72_Pd_28_	Cast	Aerated NaOH (0.01 mol dm^−3^)	−879	0.25	[104]
Al_67_Pd_33_	Cast	Aerated NaOH (0.01 mol dm^−3^)	−892	0.34	[104]
Al_70_Pd_25_Co_5_	Cast	Aerated NaCl (0.6 mol dm^−3^)	−677	0.10	[107]
Al_74_Pd_12_Co_14_	Cast	Aerated NaCl (0.6 mol dm^−3^)	−758	0.18	[107]
Al_93_Co_5_Ti_2_	Powder metallurgy sintered	Aerated NaCl (0.6 mol dm^−3^)	−707	1.1	[110]
Al_88_Co_10_Ti_2_	Powder metallurgy sintered	Aerated NaCl (0.6 mol dm^−3^)	−676	14	[110]
Al_83_Co_15_Ti_2_	Powder metallurgy sintered	Aerated NaCl (0.6 mol dm^−3^)	−490	4.0 × 10^−4^	[110]
Al_78_Co_20_Ti_2_	Powder metallurgy sintered	Aerated NaCl (0.6 mol dm^−3^)	−669	2.39	[110]
Al_73_Co_25_Ti_2_	Powder metallurgy sintered	Aerated NaCl (0.6 mol dm^−3^)	−661	0.64	[110]
Al_68_Co_30_Ti_2_	Powder metallurgy sintered	Aerated NaCl (0.6 mol dm^−3^)	−649	7.0	[110]

## Data Availability

This is a review article. The data used in this paper are publicly available in cited references.
